# 

**Published:** 1882-06

**Authors:** 


### INDEX TO VOLUME XLIV.

PAGE.

A.

Abandonment of the Spray.... 295
A Blind Horse for a Surgeon.. 111
Abstracts.................... 416
Advancing the Standard....... 492
Alienist and Neurologist, Notice.............. 144
Alumni Association of the Chicago Medical College... ... 491
American Nervousness, G. M. Beard, M.D................. 171
American Med. Association, 487 578
Anatomist, The, Review....... 622
Andrews, E., M.D., Antiseptic Surgery in the United States. 459
Andrews, E., M.D., Inefficiency of Thymol as an Antiseptic.. 461
Andrews, Dr., Chronic Carbolic Acid Poisoning, Paper...... 39
Antiseptic Surgery......... ... 107
Ankle-Joint Disease, N. M. Schaffer, M.D ............. 645
Anti-Vaccination, Consequence of ....................... 627
Antiseptic Surgery in the United States, Edmund Andrews, M.D......................... 459
Anæsthetic Mixtures.......... 372
Anatomy, Essentials of, Darling & Ranney, Review.......... 301
Aneurism, Report on, by Prof. Fenger..................... 291
Applied Anatomy of the Nervous System, Review........ 513
Artificial Feeding in Phthisis.. 630
Artificial Anæsthesia and Anæsthetics, H. M. Lyman, M.D... 175

B.

Bacillus Lepræ, The, a Reality or a Fiction, Dr. H. D. Schmidt.................. 337
Bacon and the Microscope.... 51
Bartholow, Roberts, M.D., Consumption, Treatment of..... 102

PAGE.

Bartlett, John, M.D., Forceps Blade Director.............. 361
Beard, G. M., M.D., American Nervousness........ ...... 171
Beard, R. O., M.D., Diagnosis of cause of Sudden Unconsciousness....................... 561
Belfield, W. T., Vienna Letters, 364, 493, 613
Belfield, London Letter....... 17
Bettman, B., Dr., Condition of Eyes in Pernicious Anæmia. 295
Bilroth, Theo. Prof., Ovariotomy, by.................... 284
Bigelow, Horatio M.D., Trade-Mark Pharmacy......... ... 130
Biological Society, Meeting of April 5, 1882............... 485
Bleeding from the Nose, Immediate Arrest of... ...... 56
Boracic Acid in Skin Affections. 121
Botany, Medical, of Southern Illinois, J. M. G. Carter, M.D. 132
Boston Letters.............164 498
Boone, Dr. L. D., Death of..... 170
Bone-Grafting................ 629
Books and Pamphlets Received 49, 306, 624
Bridge, Norman, M.D., Erysipelas in Childbed without Puerperal Fever................. 1
Byrd, William A., Excision of Intestinal Canal............ 598

C.

Capsicum in Metrorrhagia.... 23
Carbolic Acid Poisoning, Is there a? Dr. Andrews. Paper ....................... 39
Carter, J. M. G., M.D., Medical Botany of Southern Illinois. 132
Carpenter, W. B., M.D., The Microscope and its Revelations. 178
Carbolic Acid in Pyrexias, Raymond....................... 395

PAGE.

Carpenter, Alfred, M.D., Address on Domestic Health......... 428
Carcinoma of Tongue......... 469
Changes and Appointments.... 180
Cheatham, W., M.D., Epilepsy Cured, Etc.................. 297
Chemistry, Clowes, Review.... 298
Chemical Physiology and Pathology, Vaughan, Review... 299
Church, J. C., M.D., Death of... 315
Chemistry, General Medical, R. A. Witthaus, A.M. M.D., Review...................... 405
Chlorate of Potassium, Dr. J. V. Schoemaker............. 585
Circulation of the Blood, History of. Lecture on, by Wm. Rutherford, M.D............. 526
Clinical Reports.......... 284 468
Clinics..112, 224, 336, 448, 560, 672
Cocobacteria in Purulent Otorrhœa.................... ..... 7
Consumption, Treatment of Various Forms of, Roberts Bartholow, M.D.... ....... 102
Cold Catching, Mental Phase of, 163
Correction..................... 179
Constipation in Infants ....... 223
Contagious Diseases, Prevention of, Introduction of...... 427
Cook, W. G., Letter from Tucson ........................ 502
Cook County Comissioners, Action of ............ ..... 509
Cow-pox, New Stock of ...... 628
Cook County Hospital, Addition to Staff.................... 509
Curtis, W. H., M.D., Disposal of the Dead, Paper on......... 37
Chicago Biological Society.485, 291
Chicago Medical Society, Meetings....35, 149, 389, 472, 479, 482
Chicago Pathological Society.. 145
Correspondence, Domestic..296 619 508
Correspondence, Foreign....17 364, 495, 613, 159, 493

D.

Davis, N. S., M.D., Typhoid Fever........................ 113
Diphtheria................. 225
Dangers of Nerve-Stretching... 388
Deafness in Children ......... 512
Deafness in School Children... 492
De Quincy, Home for Inebriates.... .................. 170

PAGE.

Dentigerous Cyst ,E. S. Talbot, M.D., D.D. S .. .... .......... 143
Diagnosis of Cause of Sudden Unconsciousness, R. O. Beard, M.D ........................ 561
Dissections, Illustrations of, Review ....... .... ....... 622
Diphtheria, No Bacteria in..... 334
Diphtheria, Lecture on, N. S. Davis, M.D.................. 225
Disseminated Tuberculosis, Origin of, C. Creighton, M.D..... 208
Disposal of the Dead, Paper on, Dr. W. H. Curtis. .......... 37
Diphtheria and Treatment, Z. Magill, M.D................ 12
Domestic Correspondence..296, 378
New York Letter........... 24
Letter from A. D. Rockwell, M.D ...................... 29
Letter from H. Rosencrans, M.D ...................... 31
Letter from Los Angelos..... 373
Domestic Health, an Address on, by Alfred Carpenter, M.D. 428
Dolan, Thomas M., on Rabies of Hydrophobia, Review.... 400
Dodge, C. M., M.D., Death of... 315
Dow, L. L., M.D., Rabies, Cause and Prevention.. .......... 200
Duboisia, Action of, on the Circulation............ ...... 179
Dystocia, Peculiar Condition of Cervix Uteri in, A. Hosmer, M.D ....................... 92

E.

Editorial....... .48, 180, 409 626
Elimination of Strychnia Through the Urine.......... 510
Epilepsy Cured by Enucleation of Eye, Cheatham .......... 297
Erysipelas in Child-bed without Puerperal Fever, Norman Bridge, M.D................ 1
Essentials of the Principles and Practice of Medicine, Henry Hartshorne, Review......... 406
Ethics, Questions on, by the *Pharmacist and Chemist* .... 414
Excision of Pylorus........... 471
Eye, a Treatise on the Diseases of the, Henry D. Moyes, A.M., M.D., Review................ 403
Eye, Sympathetic Diseases of, Ludwig Mauthner, M.D., Review...................... 402

PAGE.

Eye, Injuries of, C. A. Lambert, M.D ..................... 138
Eyes, Condition of in Pernicious Anæmia.............. 298

F.

Fallopian Tubes, Dilatation and Suppuration of............. 391
Femur, Ununited Fracture of, Dr. George W. Nesbit, American Medical Association.... 592
Female Doctors in Russia. ... 388
Fenger, Chr., M.D., Report of Cases of Aneurism.......... 291
Paper on Opening and Drainage of Joints............... 149
Fisher, Dr. Alexander, Obituary ....................... 446
Fitch, W. H., M.D., Letter from Heidelberg....... ........ 368
Fitch, W. H., M.D., Vienna Letter ....................... 159
Flint, Austin, M.D., Antipyretic Treatment of Typhoid Fever, Lecture on.... ... ........ 81
Foreign Correspondence...... 17, 159, 493, 364 368
Food and Dietetics, Pavy, Review ....................... 305
Fungoid Growths, Paper on, by E. B. Stuart................ 463
Forceps Blade Director, John Bartlett, M.D ............ ... 361

G.

Gardiner, E. J., M.D., A Case of Malarial Keratitis.... ..... 14
Gay, W., M.D., Heaton's Operation for Hernia..... ... 188
German-English Dictionary... 622
Generation, a New Theory of, H. D. Valin, M.D............ 122
Gossypium Herbaceum....... 142
Gross, S. W., on Disorders of the Male Sexual Organs, Review............... ... ... 399
Guiteau Trial, Result of....... 311
Gynæcology and Obstetrics, Atkinson, review.... ........ 302

H.

Hammond on Nervous System, Review ..................... 303
Hatfield, M. P., Bemis vs. Hayes 574
Hartshorne's Essentials, Review....................... 406

PAGE.

Hagenbach, Infirmary Practice 264
Harvard Medical School, Papers of, Review............ 299
Head, Injuries of, with Remarks, J. C. Schapps, M.D... 65
Hæmorrhoids, External....... 577
Heart, Physical Diagnosis of, Review........ ........... 623
Hæmorrhoids, Hot Water in Treatment........... ..... 249
Heart, the, and its Function, Health Primer, Review...... 407
Heidelberg Letter, W. H. Fitch. 368
Hernia, Heaton's Operation for, George W. Gay, M.D........ 188
Hernia....................... 49
Histology, A Manual of, Satterthwaite, Review ......... .. 407
Histology, Practical, W. Sterling, M.D., Review.......... 176
Hollister, J. H., M.D., Typhoid Fever in Chicago....... ... 479
Hotz, F. C., M.D., Malarial Keratitis......... ............ 147
Hotz, F. C., M.D., Yellow Oxide of Mercury in Ophthalmic Practice..... .. .......... 449
Hotz, F. C., M.D., Malarial Inflammation of Middle Ear, Report of Cases........... 482
Hospital Reports.............. 442
How to Contribute to Medical Journals .................. 363
Holmes, System of Surgery, Review, Vol. I... ........... 176
Holmes, System of Surgery, Vol. II, Review............ 408
Hosmer, A., M.D., Peculiar Condition of Cervix Uteri in Some Cases of Dystocia.......... 92
Hospitals.................... 54
Holmes, Dr., Ophthalmological Cases, Two.......... ....... 40
Homœopathy, What Is It? Review.... ...... .......... 620
Homœopathy, What Is It? Reply to..................... 617
Hosmer, Alfred, M.D., on Viewing Dead Bodies in Certain Cases ............ ....... 548
Hydrocele, Radical Cure of.... 442
Hypodermic Injection of Quinine ...................... 426
Hydrophobia, or Rabies, Thos. M. Dolan, L.R.C.P., etc., Review........ ........... 400
Hysteria, Curious Case of...... 629

PAGE.

I.

Illinois State Board of Health, Examination of Candidates.. 503
Illinois State Medical Society, Review of.................. 404
Illustrations of Dissections, Ellis and Ford, Review........ 406
Impotence, Sterility and Allied Disorders of the Male Sexual Organs, by Samuel W. Gross, A.M., M.D.................. 399
Illinois State Board of Health, List of Questions to Applicants for Licenses........... 503
Ingals, E., M.D., Paper on Management of Parturition...... 41
Ingals, E., M.D., Paper on Medical Legislation.............. 607
Indiana State Medical Society, Review.................... 623
Infant Feeding and Infant Foods.....................514, 634
Intestinal Canal, Resection of, Paper ..................... 5 8
In what Cases shall the Medical Examiner Decline to View a Dead Body?...........330, 548
Insanity from Traumatism, W. P. Verity, M.D., Case.......... 484
Inefficiency of Thymol as an Antiseptic, E. Andrews, M.D.. 461
Intestinal Resection........... 221
Indian Habits and Diseases.... 180
Infancy and Childhood, Treatise on the Diseases of, J. L. Smith, M.D. ............... 176
Insane, the Rights of, Dr. C. H. Hughes, Paper.............. 604
Iodoform, Use as Surgical Dressing, Vienna Letter..... 364
Iodoform Dressing, Poisoning by....................... 363
Iodoform in Skin Diseases.... 16
Items......... .. ...217, 556 669
Items, Rules of Michigan State Board of Health............ 108

J.

Janney, Wm. S., M.D., Coroner of Philadelphia, 108 Deaths without Identity......... ... 363
Janvrin, Letter from New York 618
Jenks, Wm. T., M.D., Obituary.. 216
Journals Wanted............. 297

K.

Kane, H. H., M.D., Opium, Chloral and Hashish Habits...... 211

PAGE.

Kane, H. H., M.D., List of Questions to Practitioners......... 508
Keratitis, Malarial, E. J. Gardner, M.D..................... 14
Kiernan, Dr. James G., Simulation of Insanity of the Insane, Paper on.................. 389
The Occipital Lobe in relation to Intelligence, Paper on..... 481

L.

Lallemand, A Further Vindication of, Geo. H. Picard, A.M., M.D ......................... 3
Lambert, C. A., M.D., Cocobacteria in Purulent Otorrhœa.. 7
Lambert, C. A., M.D., Injuries of the Eye.... ............ ... 138
Laparotomy, Paper on with Discussion, American Medical Association ................. 586
Lee, Dr. John G , Paper....... 582
Letter from St. Louis. .. ..... 508
Leonard, C. H., Student's Dose-Book and Anatomist, Review, 401
Lee, E. W., Paper, Opening and Drainage of Joints.......... 149
Los Angeles, Meteorological Report of 1880....... ....... 374
Los Angeles Letter, Dr. N. Bridge ...................... 373
Librarian's Report, American Medical Association......... 604
Liver, Surgery of, Dr. Joseph Ranoshoff.................. 599
Little, James M., On a Modification of Lister's Antiseptic Dressing.................... 416
Lister's Antiseptic Dressing, James M. Little, M.D., etc.... 416
Lusk, W. T., A.M., M.D., Midwifery Art and Science of, Review......................... 402
Lyman, H. M., M.D., Anæsthesia 174
Lydston, G. F.., M.D., Pulsatilla. 261
Lyon, H. M., Oral Review of Therapeutics .............. 146
Lyman, H. M., M.D., Rupture of the Heart................... 145

M.

Management of Parturition, Paper on, by E. Ingals, M.D...... 41
Magill, Z., M D., Diphtheria and Treatment... .. ........... 12
Marsh, Howard, M.D., Caries of the Spine.................. 57

PAGE.

Malarial Keratitis, F. C. Hotz, M.D .......................... 147
Manthur, Ludwig, M.D., Sympathetic Diseases of the Eye, Review .......................... 402
Masturbation .................. 415
Malarial Inflammation of the Middle Ear, Discussion on... 482
Marcy, H. O., M.D., Address in Obstetric Section American Medical Association ....... 591
Malaria in Rhode Island...... 612
Medical Student's Primer..... 619
Medical Legislation, Contribution to Report of Committee of, E. Ingals, M.D........... 607
Meteorological Report of Los Angeles, 1880............... 374
Membranous Dysmenorrhœa... 353
Medical Examiner The, In what Cases shall he Decline to View a Dead Body.......548, 330
Mead, J. A., M.D., Death of. ... 315
Meadows, Alfred, M.D., Menstruation and its Derangements.. 183
Menstruation and its Derangement, Alfred Meadows, M.D.. 183
Meteorology and Croupous Pneumonia ................ 158
Michigan, State Board of Health of, Report, Review........... 624
Michigan, State Board of Health 489
Midwifery, Science and Art of, W. T. Lusk, A.M., M.D., Review........................ 402
Microscope, the, W. B. Carpenter, M.D..................... 178
Michigan State Board of Health. 154
Michigan State Board of Health, Rules for Prevention and Restriction of Contagious Diseases ........................ 108
Mortality in U. S. Tables ...... 670
Mother's Guide, The, Review.. 621
Monodynamics, Dr. Wright, Review ........................ 300

N.

Nasal Catarrh, Treatise on, Robinson, Review................ 304
Nerve-Stretching in Tetanus, W. Wheeler, M.D.............. 194
Neoplasm H. D. Valin, M.D.... 254
Nervous System, Treatise on the Diseases of, Hammond, Review ........................ 303
Nerve-Stretching, Dangers of,.. 388

PAGE.

Nervous Disease, New Form of, with Essay on Erythoxylon Coca, W. S. Searl, M.D., Review ........................ 401
New York State Medical Society and Ethics............ 409
New York Letter .......... 378
New York Delegates to American Medical Association..... 591
Nesbit, Geo. W., M.D., Paper on Fracture of Femur Ununited, Discussion .................. 592
Neuralgia, Differential Diagnosis ........................ 668
Nitro-Glycerine in Heart Disease ........................ 423
Night Medical Service........ 398
Notes From Foreign Societies. 390
Noyes, Henry D., A Treatise on Diseases of the Eye, Review. 403
Number of Physicians........ 392

O.

Occipital Lobe in Relation to Intelligence, Paper by Dr. J. G. Kiernan and Discussion, Chicago Medical Society..... 481
Obituary..................... 446
Obituary, Wm. F. Jenks, M.D.. 216
Octerlony, Dr. J. A., Address on Practice of Medicine, American Medical Association..... 589
Old Journals Wanted......... 297
Original Translations, Elimination of Strychnia through the Urine, F. H. Gentsch...... 510
Original Communications..... 250
Original Translations, Tuberculosis, Etiology and Prophylaxis of.................... 307
Original Translations, Austin C Ramsey.................... 393
Ophthalmic and Otic Memoranda, D. B. St. John Roosa and Edward T. Ely, Review.. 401
Ol. Sant. Flav................ 144
Opium, Chloral and Hashish Habits, H. H. Kane, M.D .... 211
Opening and Drainage of Joints, Fenger and Lee............ 149
Original Translations......... 628

P.

Paris Academy of Medicine... 217
Pathology and Morbid Anatomy, T. H. Green, M.D....... 178

PAGE.

Pasteur's Experiments on Charbon .......... 220
Pathogenetic Outlines of Homœopathic Drugs, Heinicke, Review .......... 303
Packard, John H., Sea Air and Sea Bathing, Review .......... 401
Parturient State, The Post-Partum Management of, Wm. M. Polk, M.D., etc .......... 419
Pessary Retained for Eleven Years .......... 627
Pemphigus, Leon Sandberg, Russia .......... 660
Phosphate of Lime in Bronchitis .......... 396
Physiological Laboratory, Harvard Medical College, Papers of .......... 299
Physicians and Pharmacists... 180
Pharyngeal Tuberculosis ..... 390
Picard, Geo. H. M.D., A Further Vindication of Lallemand... 3
Pirogoff, Professor, Death of... 309
Placenta An Accessory .......... 628
Polk, Prof. Wm. M., The Parturieat State .......... 419
Posological Table, by Chas. Rice, Review .......... 40
Preliminary Notice of College for Medical Practitioners, St. Louis .......... 555
Progress of Medicine, Variola. 553
Practice at the County Infirmary .......... 264
Practice of Medicine by Druggists .......... 53
Predisposing Causes of the Alcohol and Opium Habits, M. H. Thompson, M.D .......... 35
Prolific .......... 577
Psychology in Physic .......... 50
Pneumonia, Treatment at Bellevue .......... 443
Puerperal Eclampsia, Forceps Delivery and Venesection.... 386
Pulsatilla in Inflammation of the Testes, G. F. Lydston.... 261
Puerperal Eclampsia, Trumbull .......... 250
Pylorus, Excision of .......... 471

Q.

Quack Advertisements .......... 219
Quebracho .......... 222

PAGE.

R.

Rabies, Cause and Prevention, Dow .......... 200
Ramsey, A. C., Translation from *Revue Médicale*, Washing of the Stomach .......... 393
Raymond, Internal use of Carbolic Acid in Pyrexias ...... 395
Reviews and Book Notices..... 171, 298, 399, 513, 620
Recent Crimes .......... 310
Rectum, Diseases of, and the Surgery of the Lower Bowel, Review, W. H. Van Buren, M.D., etc .......... 400
Recurrent Melancholia; Persistent Attempts at Suicide by Swallowing Pins and Needles 444
Records of Vaccinations. ..... 497
Resolutions by International Medical Congress as to Tests of Sight for Signal and Lookout Men .......... 541
Rice, Letter from La Moille.... 169
Rice, Charles, Chemist, Posological Table, Review ....... 400
Ricordiana .......... 671
Rockwell, A. D., M.D., Letter from .......... 29
Rosecrans, H., M.D., Letter..... 31
Root, D. S., M.D., Death of..... 315
Root, Dr. D. S., Obituary...... 447
Rutherford, Wm., M.D., Lecture on Circulation of the Blood, History of Discoveries Concerning .......... 526
Rupture of the Heart, Paper on, H. M. Lyman, M.D .......... 145

S.

Sanitary Science .......... 54
Satterthwaite, Thos. E., M.D., Manual of Histology, Review. 407
Salicilate of Potash in Acute Rheumatism, Paper on, Dr. M. Donnelly .......... 603
Schapps, J. C., M.D., Injuries of Head .......... 65
Schmidt, Dr. H. D., Identity of the Bacillus Lepræ .......... 337
Schoemaker, Dr. J. V., Chlorate of Potassium .......... 585
Treatment of Syphilis with Subcutaneous Injections... 595
Schaffer, N. M., M.D, Ankle-joint Disease .......... 645
Sea Air and Sea Bathing, John H. Packard, Review .......... 401

PAGE.

Seasickness, Stocker, *N. Y. Med. Jour* .... 316
Selections ....428, 514, 634
Sickness in the City .... 170
Sight, Tests for in Signal and Lookout Men .... 541
Simulation of Insanity by the Insane .... 389
Sketches of Officers of Am. Med. Association .... 605
Skin, The Boracic Acid in Certain Diseases .... 121
Small Pox .... 471
Small Pox in Richmond .... 441
Smith, Dr. Protheroe .... 182
Smith, J. L., M.D., Diseases of Children .... 176
Society Meetings ....112, 336, 448
Society Reports .... 578
Society Reports .... 472
Society Reports, Chicago Medical Society .... 389
Spine, Caries of the, in Childhood, Howard Marsh, M.D. .. 57
Stuart, E. B., Nature and Affinities of Certain Fungoid Growths .... 463
Students' Dose-Book and Anatomist Combined, C. Henri Leonard, M.D., Notice .... 401
Stroinski, O., M.D., Membranous Dysmenorrhœa .... 353
Sterling, Practical Histology, Review .... 177
Stroinski, O., M.D., Double Uterus .... 10
Suicide, Paper on, Dr. John G. Lee .... 582
Sutton, R. Stansbury, M.D., Report of Ovariotomy .... 284
Syphilis, Treatment of, J. V. Shoemaker, M.D .... 595
Syphilis, Communication by Skin Grafting .... 283

T.

Talbot, E. S., Dentigerous Cyst. 143
Temperature, Low, on Exposure to Cold .... 628
Tetanus, Epidemic of .... 628
Thompson, Mary H., M.D., Alcohol and Opium Habits, Predisposing Causes of .... 35

PAGE.

Therapeutics, Progress of .... 312
Tonga Trade Mark Litigation .. 669
To the Medical Profession, as to the Code of Ethics .... 414
Too Many Doctors .... 551
Traumatic Luxation of the Foot 468
Trade Mark Pharmacy, H. R. Bigelow, M.D .... 130
Treatment of Felons .... 110
Trumbull, Theodore, M.D., Paper 250
Typhoid Fever in Chicago, Paper by Dr. J. H. Hollister .... 479
Typhoid Fever in Cook County Hospital, H. D. Valin, M.D. .. 286
Typhoid Fever, N. S. Davis, M.D. 113
Typhoid Fever, Antipyretic Treatment of, Austin Flint, M.D .... 81

U.

Urinary Organs, Surgical Disorders of, Harrison, Review .. 305
Uterus, Double, O. Stroinski, M.D .... 10
U. S. Pharmacopœa, Revision of 625

V.

Vaccination, Does it Protect? .. 52
Valin, H. D., M.D., Paper on A New Theory of Generation .. 122
Paper on Neoplasm .... 254
Vertebrate Dissection, Martin and Moale, Review .... 300
Variola .... 553
Vaccinations, Records of .... 497
Van Buren, On Diseases of the Rectum, Review .... 400
Vienna Letter, W. H. Fitch, M.D. 159
Vienna Letters, W. T. Belfield, M.D ....364, 493, 613
Vivisection .... 50

W.

Wheeler, W. J., M.D., Successful Nerve Stretching in Tetanus. 194
Wilderness Cure, The, Cook, Review .... 301
Witthaus, R. A., A.M., M.D., General Medical Chemistry, Review .... 405
Woman's Medical College, Baltimore .... 408

### BOOKS AND PAMPHLETS RECEIVED.

Foster's Physiology. 2d Edition. H. C. Lea.

Dalton's Physiology. 7th Edition. H. C. Lea.

Sensation and Pain. Taylor. G. P. Putnam's Sons.

Physicians' Clinical Record. D. G. Brinton. Philadelphia.

Winter and its Dangers. Osgood. P. Blakiston.

The Anatomist. Hilles. G. P. Putnam's Sons. New York.

Opium Smoking. Kane. G. P. Putnam's Sons.

Eczema and its Management. Bulkley. G. P. Putnam's Sons.

Holmes' System of Surgery. Vol. III. H. C. Lea's Son & Co. Philadelphia.

Diseases of the Chest, Throat and Nose. Ingals. Wm. Wood & Co. New York.

A New Form of Nervous Disease. Searl. Fords, Howard & Hurlbut.

The Nurse and Mother. Coles. J. H. Chambers & Co. 1881.

Illustrations of Dissections. Ellis and Ford. Wm. Wood & Co. New York.

Handbook on Diseases of Women. Brown. Wm. Wood & Co.

State Board of Health, Massachusetts. 1880. Rand, Avery & Co. Boston.

Diseases of Children. Meigs and Pepper. Blakiston, Son & Co.

Index of Surgery. Keetley. William Wood & Co.

Treatment of Varicocele. Henry. Vail & Co. New York.

Lectures on Diseases of Women. Henoch. Wood & Co. New York.

Coleman's Dental Surgery and Pathology. Stellwagen. Lea. Philadelphia.

Materia Medica and Therapeutics. Inorganic Substances. Phillips. L. Johnson. New York.

The Incidental Effects of Drugs. Lewin. Wm. Wood & Co.

Transactions of American Medical Association, 1881. Collins. Phila.

Physiological Chemistry of the Animal Body. Gamgee. MacMillan & Co. London.

Diseases of the Eye. Williams. Houghton, Mifflin & Co. Boston.

The Physician Himself. Cathell. Cushings & Bailey. Baltimore.

---

THE U. S. PHARMACOPŒIA.—The revision of the *Pharmacopœia*, we are informed, is nearly completed. The work will make a duodecimo volume of from 400 to 450 pages. The mode of issuing it must be very soon considered and decided. The copyright will be disposed of, no doubt, in accordance with the instructions, on the best terms obtainable. The manner of inviting proposals for the publication of the work has not yet been determined. It is probable, however, that those who think of competing for the copyright may obtain the information desired by applying to the chairman of the "Committee of Revision," Mr. Charles Rice, Bellevue Hospital, New York.—*Druggists' Circular.*

CLINICS.

MONDAY.

Eye and Ear Infirmary—1.15 p. m., Otological, by Prof. Jones; 2.15 p. m., Ophthalmological, by Prof. Hotz.

Mercy Hospital—2 p. m., Medical, Profs. Hollister and Quine.

Rush Medical College—3 p. m., Dermatological and Venereal, by Prof. Hyde.

Woman's Medical College—2 p. m., Dermatological and Venereal, by Prof. Maynard.

TUESDAY.

Cook Co. Hospital—2 to 4 p. m., Medical and Surgical Clinics.

Mercy Hospital—2 p. m., Surgical Clinic, by Prof. Andrews.

Woman's Medical College—10 a. m., Prof. Ingals.

WEDNESDAY.

Chicago Medical College—2 p. m., Eye and Ear, by Prof. Jones.

Rush Medical College—2 p. m., Medical, by Dr. Bridge; 3 p. m., Ophthalmological and Otological, by Prof. Holmes; 3:30 to 4:30 p. m., Diseases of the Chest, by Dr. E. Fletcher Ingals.

Woman's Medical College—2 p. m., Eye and Ear, by Dr. W. T. Montgomery.

Eye and Ear Infirmary—2.30 p. m., Dr. E. J. Gardiner.

THURSDAY.

Chicago Medical College—2 p.m., Gynæcological, Prof. Dudley.

Rush Medical College—2 p. m., Diseases of Children, by Dr. Knox; 3 p. m., Diseases of the Nervous System, by Prof. Lyman.

Eye and Ear Infirmary—2 p. m., Ophthalmological, by Dr. Hotz.

Woman's Medical College—3 p. m., Surgical, by Prof. Owens.

FRIDAY.

Cook County Hospital—2 to 4 p. m., Medical and Surgical Clinics, by Profs. J. H. Hollister and R. N. Isham.

Mercy Hospital—2 p. m., Medical, by Prof. Davis.

SATURDAY.

Rush Medical College—2 p. m., Surgical, by Prof. Gunn.

Mercy Hospital—2 p. m., Surgical Clinic, by Prof. Andrews.

Chicago Medical College—3 p. m., Neurological, Prof. Jewell.

Woman's Medical College—2 p. m., Gynæcological, by Prof. Stevenson.

Daily Clinics, from 2 to 4 p. m., at the Central Free Dispensary, and at the South Side Dispensary.

